# Impact of Patient’s Age and Physician’s Professional Background on the Number Needed to Treat in Malignant Melanoma Detection

**DOI:** 10.3390/cancers16234014

**Published:** 2024-11-29

**Authors:** Laura Schreieder, Veronika Zenderowski, Mark Berneburg, Sebastian Haferkamp, Konstantin Drexler, Dennis Niebel

**Affiliations:** Department of Dermatology, University Hospital Regensburg, Franz-Josef-Strauss Allee 11, 93053 Regensburg, Germany; veronika.zenderowski@ukr.de (V.Z.); mark.berneburg@ukr.de (M.B.); sebastian.haferkamp@ukr.de (S.H.); konstantin.drexler@ukr.de (K.D.); dennis.niebel@ukr.de (D.N.)

**Keywords:** nevus, melanoma, number needed to treat, number needed to excise, statutory skin cancer screening, dermatologic oncology

## Abstract

**Background/Objectives:** With regard to excision of pigmented lesions for detection of malignant melanoma (MM), the number needed to treat (NNT) describes the number of melanocytic nevi that need to be biopsied/excised to detect one MM. The aim should be a low NNT. **Methods**: Single-center data analysis, including dermatohistopathological records of all nevi and MM cases during 2004–2013 at the Department of Dermatology, University Hospital Regensburg (UKR), was performed. We calculated the NNT, correlating it with the patient’s age and referring physician. The MM to MM in situ ratio was calculated to quantify early detection. As a secondary objective, we stratified into a pre- and post-2008 dataset, coinciding with the introduction of statutory skin cancer screening in Germany. **Results**: The overall NNT of 118,668 pigmented lesions was 17.2. We found a linear decrease in NNT towards older patients (R^2^ = 62%; *p* < 0.001). The impact of skin cancer screening in 2008 was marked by a reduction in biopsies/excisions, a shift in age distribution, and a decrease in the NNT from 20.3 to 14.7. Office-based dermatologists had an NNT of 22.3, UKR-based dermatologists had an NNT of 8.0, and non-dermatologists had an NNT of 16.5. **Conclusions**: The age-related decrease in the NNT emphasizes the importance of age stratification for pigmented lesions. The NNT differed between professional settings. The implementation of skin cancer screening in 2008 was associated with a reduced NNT.

## Simple Summary

In the assessment of pigmented lesions, it is crucial to avoid unnecessary procedures while ensuring that melanomas are not missed. This study aims to understand how the number needed to treat (NNT) in melanoma detection varies with a patient’s age and the type of referring physician. By analyzing data from the University Hospital Regensburg, we found that the NNT decreased with advancing age. The type of referring physician was also significant: dermatologists from university settings had the most accurate referrals, requiring fewer procedures compared to office-based dermatologists and non-dermatologists. Additionally, the introduction of Germany’s skin cancer screening program in 2008 significantly reduced the NNT. These findings may help refine melanoma detection strategies and improve screening practices in the future.

## 1. Introduction

The concept of the number needed to treat (NNT) serves as a pivotal measure for evaluating the effectiveness of treatments and interventions, particularly in the context of the introduction of new diagnostic tools. For the diagnosis of malignant melanoma (MM), the NNT can be used to define how many pigmented lesions that are suspected to be malignant by the assessing physician need to be excised or biopsied to detect one MM [1]. The NNT is calculated by dividing the cases of all biopsies and excisions with the diagnoses of MM and is influenced by diagnostic accuracy and disease prevalence. The main aim should be to minimize unnecessary procedures, promote the responsible use of healthcare resources, and thereby reduce the economic burden while avoiding missing or overlooking skin cancers, including MM [2,3]. Unnecessary biopsies and excisions have the potential to cause anxiety in patients [4] and carry the risk of wound healing problems, infection, nerve damage, and tissue defects [5,6]. These complications further emphasize the need for precision in diagnostic approaches.

To reduce MM-related morbidity and mortality, early detection and excision of the entire lesion is the primary strategy. Timely identification of MM in its early stages, characterized by a thin primary tumor or, even better, in situ lesions, correlates positively with a favorable prognosis [7]. MMs have been found to be thinner when diagnosed through skin cancer screening [8,9,10,11]. However, it is important to acknowledge that not all early-stage MMs pose a significant risk to the patient’s survival, and the impact of early detection on MM-specific mortality remains a topic of debate. In addition, early detection has been associated with a reduction in consultations per patient to office-based physicians and clinics, thereby reducing the burden on the healthcare system [12]. In contrast, the management of metastatic disease often remains a challenge with a significantly worse prognosis that has shown only slight improvement despite therapeutic advances in recent years [13,14,15]. 

In July 2008, the German Federal Ministry of Health instituted a resolution for a nationwide statutory skin cancer screening. This directive gives people aged 35 years and older the right to a biennial total body skin examination (TBSE), a visual inspection of the entire body, including the scalp, mucosa, and the genital area, to detect lesions that may indicate skin cancer. Germany is the only European country to offer such extensive screening as a health insurance benefit [16].

The primary focus of the current study was to analyze the NNT for the detection of MM depending on variables such as a patient’s age and background of the referring physician. In addition, this study aimed to evaluate the impact of the implementation of statutory skin cancer screening in Germany in 2008 as a secondary objective. Finally, this study assessed the time point of MM detection, quantified as the ratio of invasive MM to MM in situ (MM:MMis).

## 2. Materials and Methods

Data for this study were obtained from the routine dermatohistopathological database of the Department of Dermatology at the University Hospital Regensburg (UKR) for the years 2004 to 2013. Every histopathological sample of a patient, along with their data, diagnosis, and the referring physician who performed the excision, is recorded in this registry. The search parameters “nevus” and “melanoma” regarding the final diagnosis yielded 119,378 cases. After eliminating 698 duplicates and 12 incomplete records, 118,668 cases of biopsied or excised melanocytic nevi and MMs were included in this study. 

Key patient characteristics (date of birth, diagnosis, date of diagnosis, and referring physician) were systematically recorded. This information was used to calculate the patient’s age at diagnosis. In addition, referring physicians were categorized into (1) non-dermatologists (such as general practitioners, surgeons, and other clinical specialists), (2) office-based dermatologists, and (3) UKR-based dermatologists. The dataset was further stratified into periods before and after the introduction of statutory skin cancer screening in Germany in 2008. A sensitivity analysis was conducted to assess the potential impact of including or excluding patients under 35 years of age, as they are not typically eligible for routine screening under current guidelines.

The NNT (or better: number needed to excise) was calculated for the whole dataset, each age, and calendar year, as well as for different referring physicians. The reciprocal of the proportion of melanomas among all nevi and melanomas included was calculated, meaning 1 divided by the proportion of melanoma cases to the total number of cases. The confidence intervals for the NNT were calculated.

(1) $$\frac{1}{\frac{melanoma\;cases}{cases\;of\;melanocytic\;nevi+melanomas}}$$

To assess the relationship between the NNT and a patient’s age as well as calendar year, we employed a stepwise modeling approach. Initial analyses used a simple linear regression to evaluate a potential linear association. Given the exponential trend observed in the scatterplot, the dependent variable NNT was logarithmically transformed to address non-linearity. Diagnostic evaluations (e.g., residual plots, Q-Q plots) were conducted to check model assumptions, including linearity, homoscedasticity, and the influence of outliers. To address violations of these assumptions, a robust regression model for assessing the correlations between the NNT and patient’s age was employed, which reduces the influence of outliers using Huber weighting. Based on these findings, the robust regression model was selected as the final analytical approach, providing the most reliable estimates and addressing the key issues identified in prior models while adhering to the assumptions of regression analysis. For evaluating the trend between the NNT and calendar year, a log-linear regression model was applied, with the natural logarithm of the NNT as the dependent variable. This approach addressed slight violations of linearity observed in the initial linear regression. The goodness of fit for each model was assessed using adjusted R^2^.

The ratio of MM:MMis was calculated to assess the effectiveness of early detection of MM. Due to deviations from linearity and non-normality of residuals, a Spearman correlation analysis was employed to evaluate the monotonic relationship between age and the MM:MMis ratio. Differences in the MM:MMis ratio with respect to screening and physician profession were analyzed using Fisher’s exact test. The effect of screening on the number of excisions and the NNT was assessed using the Mann–Whitney U test.

Statistical analyses were performed, and figures, such as bar graphs and scatter plots, were generated by using the statistical software package IBM SPSS Statistics 25 (IBM SPSS, Chicago, IL, USA).

## 3. Results

### 3.1. Demographics of the Study Population

Between 2004 and 2013, a comprehensive analysis of dermatohistopathological records yielded 118,668 cases of biopsied/excised nevi and MMs. These cases formed the basis of our study, encompassing 111,767 nevi (94.2% of all cases) and 6901 MMs (5.8% of all cases).

**Figure 1:**
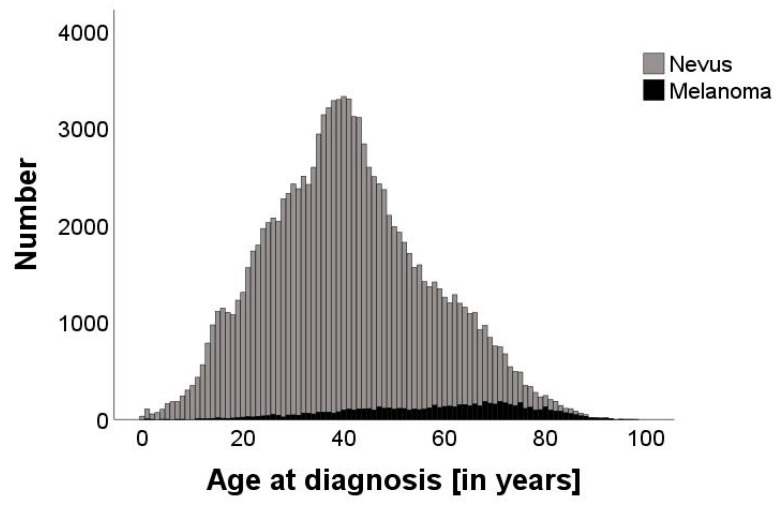
Bar graph displaying the number of nevi (grey, n = 111,767) and malignant melanomas (black, n = 6901) at different ages, ranging from 0 to 98 years. The age with the highest number of excised pigmented lesions was 40. The median age of diagnosis of nevi is 39, while the median age of diagnosis of melanoma is 61.

The age range of melanoma diagnosis varied from a minimum of 1 year to a maximum of 98 years, highlighting the diverse age distribution within our dataset. Melanocytic nevi were observed from birth to 95 years of age. Peaks in nevus occurrence were observed at 39 years of age, while MMs had the highest occurrence at 61 years of age (Figure 1). Of the MM cases, 1560 were identified as MMis, of which 303 were specifically classified as lentigo maligna.

In the context of these findings, the calculated NNT for all cases from 2004 to 2013 was 17.2 [95%-CI: 16.7;17.6]. The overall MM:MMis ratio was 3.4.

### 3.2. Correlation of the NNT with Age

**Figure 2:**
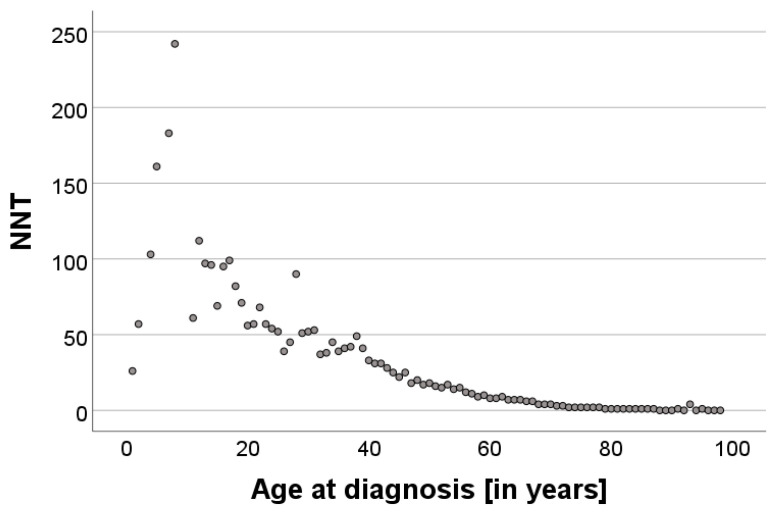
Scatterplot showing the exponentially decreasing trend of the NNT with advancing age, indicating that the proportion of nevi to melanoma decreases in older age.

Calculation of the NNT correlating with age at diagnosis showed an exponentially decreasing curve in the scatterplot (Figure 2). To address this non-linearity, the dependent variable NNT was log-transformed. The log-linear regression model indicated a strong fit (adjusted R^2^ = 90.9%) and revealed a significant negative association between age and ln(NNT) (*p* < 0.001). However, diagnostic evaluations still indicated issues with heteroskedasticity and the influence of outliers. The robust model confirmed a significant negative association between age and ln (NNT) (*p* < 0.001; β = −0.061; 95% CI: [−0.064;−0.058]). Each additional year of age corresponded to an approximately 5.9% decrease in the NNT. Diagnostic plots showed improved residual distribution and reduced heteroskedasticity compared to the log-linear model.

The MM:MMis ratio shows a significant negative monotonic relationship with advancing age, indicating an increased proportion of MMis among older patients (*p* < 0.001, Spearman-r = −0.417). This aligns with an increased prevalence of lentigo maligna in older patients [17].

### 3.3. Variability in the NNT Based on the Professional Background of the Referring Physician

Referring physicians were categorized into three groups based on their setting (office-based dermatologists, UKR-based dermatologists, and non-dermatologists).

Office-based dermatologists accounted for the majority of referrals for suspicious pigmented lesions, with a total of 98,129 cases, resulting in an NNT of 22.3 [95%-CI: 22.26;22.32], while dermatologists from the Department of Dermatology at the University Hospital of Regensburg (UKR-based dermatologists) contributed 19,498 cases, yielding an NNT of 8.0 [95%-CI: 7.96;8.04]. Non-dermatologists, including general practitioners, surgeons, and other clinical professionals, referred the fewest cases of excisions and biopsies (1025 cases) with an NNT of 16.5 [95%-CI: 16.29;16.77]. The assessment of excised and biopsied nevi and MMs therefore revealed significant differences in the NNT depending on the background of the referring physician.

In addition, the MM:MMis ratio was calculated for each group both before and after the implementation of statutory skin cancer screening in 2008, revealing a significant reduction. Specifically, the MM:MMis ratio for UKR-based dermatologists decreased from 6.1 (2004–2008) to 4.4 (2009–2013), similar to that of office-based dermatologists, whose ratio decreased from 3.7 to 2.3 over the same periods. MM:MMis ratio for non-dermatologists yielded 3.8 before and 23 after screening implementation, while only contributing one MM in situ diagnosis after. Fisher’s exact test revealed significant differences in the distribution of MM to MM in situ across all groups before and after screening (OR = 0.648; *p* < 0.001).

### 3.4. Impact of Statutory Skin Cancer Screening on the NNT

The introduction of statutory skin cancer screening in Germany in 2008 coincided with a significant reduction in the total number of excisions and biopsies, which declined from 62,112 to 56,556. At the same time, a notable shift in the age distribution was observed. After 2008 the proportion of excisions performed on patients aged 35 years and older increased by approximately 8% (Chi-quadrat test; *p* < 0.001; χ^2^ = 1610.58).

**Figure 3:**
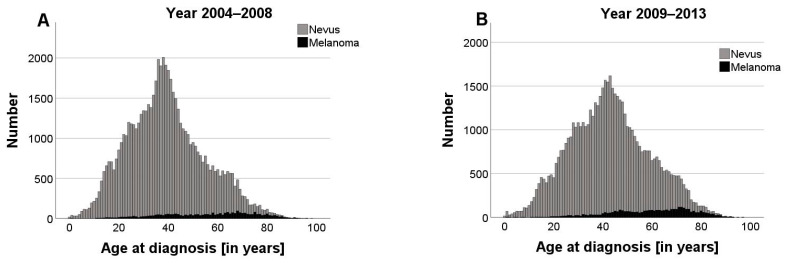
Bar graph displaying the number of diagnoses of nevi (grey) and malignant melanoma (black) at different ages before (**A**) and after (**B**) introduction of statutory skin cancer screening in Germany in 2008. The total number of excisions declined (2003–2008: n = 62,112; 2009–2013: n = 56,556), with a shift towards elderly patients. Comparing the periods before and after implementation of the screening, the median age at diagnosis increased by 3 years for nevi (38 to 41 years) and melanomas (59 to 62 years) (*p* < 0.001).

The comparison of the periods before and after the introduction of statutory screening showed a significant increase in the median age at diagnosis of nevi and MMs while the age range from 0 to 98 years remained the same. Specifically, the median patient age increased by 3 years to 41 years for nevi (2004–2008: 38 years) and to 62 years for MM (2004–2008: 59 years) (Figure 3A,B). A descriptive analysis of annual age distributions (median, 1st, and 3rd quartiles) indicated that these changes occurred gradually rather than abruptly around 2008. While the year-by-year differences are not always statistically significant, a comparison of pre-screening (2004–2008) and post-screening (2009–2013) periods showed a statistically significant shift in age distribution for MMs (Mann–Whitney U-test, *p* < 0.001; U = 5,311,399).

After the implementation of skin cancer screening, the overall calculated NNT showed a significant reduction from 20.3 [95%-CI: 20.27;20.33] to 14.7 [95%-CI: 14.69;14.75] (Mann–Whitney U-test, *p* = 0.008, U = 15,000), and the MM:MMis ratio declined from 4.4 to 2.9. Even when only considering people 35 years and older (as those under 35 years are not regularly eligible for routine screening), the NNT declined from 14.4 [95%-CI: 14.38;14.46] to 11.06 [95%-CI: 11.03;11.09]. This analysis was conducted to ensure the robustness of the results.

**Figure 4:**
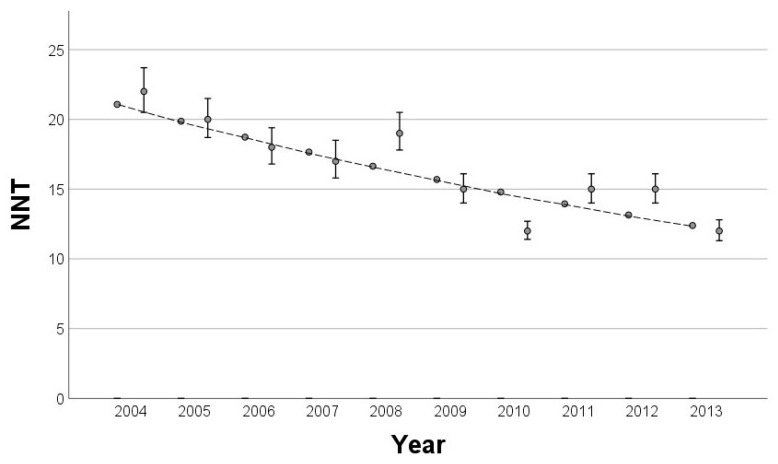
Observed NNT values with 95% confidence intervals (dots with error bars) and the fitted values based on the log-linear regression model (dashed line) over the years 2004 to 2013. The log-linear regression demonstrates a consistent decline in the NNT over time (*p* < 0.001, adjusted R^2^ 72.4%).

The analysis involved calculating the NNT for each year between 2004 and 2013. Initially, a simple linear regression model revealed a significant decline in the NNT over time (*p* < 0.001; adjusted R^2^ of 75.0%), but diagnostic plots indicated slight violations of the linearity assumption. A log-linear regression model was subsequently applied, which provided a better fit to the data by meeting key regression assumptions, despite a slightly lower adjusted R^2^ of 72.4% (Figure 4). Notably, the NNT showed a decrease of approximately 5.7% with each year.

## 4. Discussion

The main objective of this study was to investigate potential correlations between the NNT and age as well as the professional background of the referring physician. Due to the large number of cases of biopsied/excised melanocytic nevi and MMs, coupled with a diverse age distribution within our dataset, this study provides realistic insights into melanoma detection and diagnostic accuracy. In addition, the inclusion of a wide range of referrers, including 31 surgeons, 10 general practitioners, and 29 specialists from various medical fields such as otorhinolaryngologists and gynecologists, as well as 61 office-based dermatologists and the UKR-based dermatologists, who comprise an ever-changing mix of practitioners and consultants, reflects a comprehensive picture of the daily routine of MM detection. To the best of our knowledge, this is the first study to analyze the NNT of MMs in Germany in relation to the introduction of statutory skin cancer screening.

The calculated NNT of 17.2 for all cases from 2004 to 2013 is comparable to other national and international studies on this topic, which encompass a range from 4 to 40, depending on the characteristics considered [2,3,18,19,20,21,22,23,24,25]. These characteristics included age, sex, socioeconomic background, site of the lesion, profession and level of experience of the referring physician, consideration of seborrheic keratosis, and the type of screening (lesion-directed vs. TBSE). While the NNT values span a wide range, these differences are likely influenced by contextual variations, including differences in healthcare systems, physician education, skin type distribution, and local prevalence of certain skin conditions, and should be kept in mind when interpreting the results.

Our results showed a strong negative correlation with age. This phenomenon may be mainly explained by the rising incidence of MM and the decreasing number of melanocytic nevi in older people [26]. The peaks of nevus diagnosis at 39 years of age and MM at 61 years further demonstrate the distinctive age-related pattern of pigmented lesions, which is consistent with the reported incidence of MM [19,27,28]. In addition, it is plausible that there is a higher threshold for biopsy or excision of pigmented lesions in older patients, as the focus often shifts toward quality-of-life and wound-healing considerations, and physicians may opt for monitoring over invasive procedures. In contrast, younger patients tend to face a lower threshold for interventions, reflecting a proactive approach to address potentially aggressive lesions early in their longer expected lifespan and cumulative risk of progression [3,20]. Furthermore, the possibility that younger people are more health conscious and therefore request more excisions for safety reasons may contribute to this trend. These findings underline the age-related reduction in the NNT and emphasize the importance of incorporating age-related considerations into the diagnosis of MM, suggesting that screening initiatives may have a greater outcome benefit in older age groups.

This analysis reveals significant differences in the NNT based on the professional background of the referring physician. Office-based dermatologists, who contributed to the majority of cases, had a higher NNT (22.3) than UKR-based dermatologists (8.0) [22,29]. This discrepancy is largely due to the preselection of patients treated at the UKR, which requires referral from general practitioners or office-based dermatologists. The referral pattern may result in university hospitals handling more complex cases, potentially increasing the likelihood of identifying true positive cases and reducing the NNT. In addition, university hospitals may offer opportunities for second opinions from experts in melanoma diagnosis, contributing to increased diagnostic accuracy and a potential reduction in the NNT. Office-based dermatologists manage a higher volume of routine cases due to their referral pattern and may perform excisions for reasons other than suspected melanoma, resulting in a higher NNT.

Therefore, one limitation of this study is the lack of information on the reason for excision/biopsy. Non-dermatologists, despite referring fewer cases, have an intermediate NNT of 16.5, which is comparable to that of other studies [3,24]. This observation may be due to the practice among general practitioners to conduct excisions primarily in highly suspicious cases. Surgeons, in turn, may receive referrals from office-based dermatologists who lack training in dermatologic surgery. The differences in the NNT among the assessing professions can therefore be attributed to differences in the populations served as well as different levels of expertise.

The implementation of statutory skin cancer screening in Germany in 2008 correlated with a significant reduction in the overall NNT, which decreased from 20.3 to 14.7. This observed trend may be attributed to a marked decrease in the total number of excisions and biopsies, which may be a consequence of the stringent requirements for participation in skin cancer screening introduced by the German Federal Ministry of Health in 2008. These criteria required practitioners conducting skin cancer screening to be licensed dermatologists or non-dermatologists with specialized training in the clinical detection of skin cancer. Better-trained examiners are more adept at distinguishing benign lesions from malignant ones, resulting in a more efficient screening process and a lower NNT [16].

Following the introduction of skin cancer screening, there was a notable shift in the number of excisions towards patients aged 35 years and older. Moreover, the median age at diagnosis of both nevi and MMs increased by 3 years each (nevi: 38 to 41; MMs: 59 to 62). These findings suggest that the introduction of screening contributed to a shift in the age distribution. However, the change appears to be part of a broader, gradual trend rather than an isolated, immediate effect of the screening introduction. The demographic change in Germany may contribute to part of this trend, but it only added approximately 0.2 life years per year on average between 2004 and 2013 [30]. The shift is also likely to be influenced by the fact that health insurance coverage for skin cancer screening is limited to people aged 35 years and older [16], which encourages a more targeted approach focused on those with a higher likelihood of developing skin cancer, thereby reducing the NNT [16,28]. The observed trend may be attributed to the time-consuming nature of TBSE, potentially encouraging a more selective and conservative approach to excision. Patients flagged through screening could be subjected to follow-ups, allowing physicians to monitor lesions over time rather than resorting to invasive interventions. Increasing participation rates, ranging from approximately 28% in 2011 to 35% in 2013 among people aged 35 and older, may be taking up more consultation time at dermatology appointments [31,32].

Interestingly, studies suggest that lesion-directed screening (LDS) may yield MM detection rates comparable to those of TBSE, with the advantage of being less time-consuming and more cost-effective [33,34]. However, TBSE remains advisable if the index lesion in LDS is suspicious [4]. It should be noted that TBSE may result in unnecessary excisions while having higher rates of false-positive findings [4,33,35]. Subsequently, awareness of skin cancer recognition in the general population may optimize preselection. The results of LDS and risk factor-based screenings in comparison to TBSE need to be further investigated to optimize skin cancer screening. However, the responsible use of healthcare resources should also be taken into account, as Krensel et al. demonstrated that screening adds substantial costs to overall treatment expenses [36]. To assist physicians in optimizing sensitivity and specificity, an increasing number of AI-assisted diagnosis systems and app-based skin cancer screenings are anticipated in the future [37]; such tools were not available at the time of our study. However, advancements in technology and knowledge, particularly in dermoscopy, may indeed have contributed to the decrease in the NNT over the years, which likely enhanced the accuracy of MM detection. In our cohort, skin cancer screening was associated with an improvement in the overall NNT. However, this observation is based on data from our institution and cannot be generalized to all settings.

To adequately capture the diagnostic sensitivity and the additional ability to detect early-stage disease, it is essential to consider the MM:MMis rate: a lower rate signifies a higher proportion of MM in situ, indicating early detection. The decreasing trend in the MM:MMis rate with advancing age is primarily attributed to a higher prevalence of lentigo maligna among older people [17,38,39,40]. After the implementation of skin cancer screening, the MM:MMis ratio halved to 2.9, indicating improved early detection and highlighting the impact of this preventive measure, consistent with the finding that the incidence of MM in situ and T1-melanoma increased by 69% after 2008 [41,42].

Screening initiatives may have increased skin cancer awareness in the general population, resulting in patients consulting a dermatologist earlier. The effectiveness of statutory skin cancer screening is still strongly debated, as studies suggest that skin cancer screening did not show a positive effect on mortality between 2008 and 2013 [43,44,45]. In addition, the MM:MMis ratio was assessed for each professional group both before and after statutory skin cancer screening, revealing a significant reduction. Notably, the university hospital had a MM:MMis ratio twice as high as that of office-based dermatologists (4.4 vs. 2.3). This difference may be due to referrals to university hospitals for excision of thicker MMs, thereby assuming responsibility for the subsequent management of the advanced disease. In contrast, office-based dermatologists act as a preselection filter, predominantly catering to community populations and probably engaging in more routine skin checks.

## 5. Conclusions

In our center, the NNT to detect one MM in biopsies and excisions of pigmented lesions was 17.2 between 2004 and 2013. This falls within the range reported in other studies (NNT range: 4–40), indicating that our findings align with previously observed variability in detection efficiency [2,3,19,20,21,22,23,24,25,29]. The linear decrease in the NNT with advancing age indicates that age-related considerations should be incorporated into the assessment and management of pigmented lesions. Variations in the NNT were observed according to the professional background and clinical setting, with office-based dermatologists having a higher NNT than UKR-based dermatologists, whereas non-dermatologists had an intermediate NNT. The implementation of skin cancer screening in 2008 significantly reduced the NNT, influenced by fewer excisions and a higher median age at diagnosis. The MM:MMis ratio also decreased with age and halved after the implementation of screening, further emphasizing the preventive effect of screening measures.

In summary, this study provides insight into age-related factors in melanoma detection and variations among assessing professions while also highlighting the impact of skin cancer screening in our cohort. Taking the dynamics of the NNT into account, our findings may help tailor screening strategies for more precision in early melanoma detection and approach to excision, thereby improving patient care and outcomes. Further investigation regarding different screening approaches is warranted, as a limitation of our study is the single-center nature of this investigation.

## Data Availability

The data that support the findings of this study are available from the corresponding author upon reasonable request.
